# Pegylated Liposomal Doxorubicin Versus Epirubicin as Adjuvant Therapy for Stage I–III Breast Cancer

**DOI:** 10.3389/fgene.2021.746114

**Published:** 2021-09-20

**Authors:** Wenxian Hu, Kezhen Lv, Rongyue Teng, Jida Chen, Chenpu Xu, Lidan Jin, Yongxia Chen, Wenhe Zhao

**Affiliations:** ^1^Department of Surgical Oncology, Sir Run Run Shaw Hospital, College of Medicine, Zhejiang University, Hangzhou, China; ^2^Department of Breast Center, The First Affiliated Hospital, College of Medicine, Zhejiang University, Hangzhou, China

**Keywords:** breast cancer, pegylated liposomal doxorubicin, epirubicin, adjuvant therapy, efficacy and toxicity

## Abstract

**Background:** Conventional anthracyclines, like epirubicin, are cornerstone drugs for breast cancer treatment of all stages, but their cumulative toxicity could cause life-threatening side effects. Pegylated liposomal doxorubicin (PLD), an effective anti-breast cancer drug, has lower toxicity than conventional anthracyclines. This retrospective study compared the efficacy and toxicity profiles between PLD and epirubicin as adjuvant therapy for breast cancer.

**Patients and Methods:** A total of 1,471 patients diagnosed with stage I–III breast cancer between 2000 and 2018 were included in this study, among which 661 were treated with PLD and 810 with epirubicin, with 45.9 months as the median follow-up time. Anti-breast cancer efficacy was assessed with overall survival (OS) and disease-free survival (DFS), while cardiac toxicity was assessed with left ventricular ejection fraction (LVEF) and electrocardiogram (ECG).

**Results:** The Kaplan–Meier method and Cox proportional hazards model revealed that there was no statistical difference in OS or DFS between patients treated with PLD and epirubicin, regardless of cancer stages or molecular subtypes (all p-values > 0.05). In addition, patients had significantly better LEVF and ECG data after adjuvant therapy with PLD (both p-values < 0.05).

**Conclusion:** Based on the large sample size and the long follow-up time of this study, we conclude that PLD has a similar anti-breast cancer efficacy as epirubicin while inducing lower level of cardiac toxicity in Han Chinese. This study suggests that PLD-based adjuvant chemotherapy could be a better option than epirubicin for breast cancer patients especially with existing cardiac disease.

## Introduction

Breast cancer, the most commonly diagnosed cancer in women, has posed a major threat to women’s health globally. In 2018, approximately 2 million women were diagnosed with breast cancer and over 0.6 million women died of breast cancer (Ferlay et al., 2018). Due to the development of better diagnostic tools, therapeutic agents, and surgical techniques, the outcome of breast cancer has been significantly improved in the past decades (Smittenaar et al., 2016). The use of more powerful chemotherapy agents has markedly increased the survival rate of breast cancer patients, but the resulting toxicities and side effects have become a serious burden. For example, anthracyclines-based chemotherapy could provoke certain acute side effects such as vomiting, nausea and, more severely, congestive cardiac failure (Hortobagyi and Buzdar, 1993; Ryberg et al., 2008).

Doxorubicin and epirubicin are two cornerstone anthracycline drugs used to treat breast cancer of all stages (Khasraw et al., 2012). In spite of its anticancer effects, doxorubicin has showed high toxicity in a large number of studies, and cumulative use of this drug could lead to irreversible cardiomyopathy or liver damage (Mitra et al., 2001; Greish et al., 2004). Epirubicin (a 4′-epimer of doxorubicin) has a similar efficacy as doxorubicin in breast cancer treatment while its dose-dense clinical trials reveal safety comparable to that of doxorubicin (Dang et al., 2008; Nieto et al., 2010). These two drugs have noticeable difference between their toxicity profiles (particularly with respect to cardiotoxicity), and accumulated evidence shows that the use of epirubicin generally brings less side effects compared with doxorubicin, which leads to gradually decreased use of doxorubicin in recent years especially in China (Perez et al., 1991; Khasraw et al., 2012).

Pegylated liposomal doxorubicin (PLD) is formed by incorporating doxorubicin into polyethylene glycol-coated liposomes (Duggan and Keating, 2011). Pegylated liposomal doxorubicin reduces the plasma levels of free doxorubicin and alleviates the toxicity to healthy tissues while maintaining antitumor effects of doxorubicin (Gabizon et al., 2008). Previous studies have shown that PLD has a much longer half-life than that of doxorubicin (73.9 h vs. 10 min), which allows increased uptake of PLD liposomes by tumors and enhanced antitumor effects (Gabizon et al., 2016). The efficacy and safety of PLD-based adjuvant therapy has been investigated in a limited scale for breast cancer patients in previous studies (Lien et al., 2014; Zhao et al., 2017).

The aim of this current study is to compare the efficacy and toxicity profiles between PLD and epirubicin as adjuvant therapy for stage I–III breast cancer. Based on the large number of patients and their clinical data collected at Sir Run Run Shaw Hospital (affiliated with Zhejiang University, Hangzhou, China), we are able to perform a comprehensive evaluation of PLD and epirubicin in different breast cancer stages and molecular subtypes. We find that PLD and epirubicin bring similar overall survival (OS) and disease-free survival (DFS) in stage I–III breast cancer while the toxicity of PLD is considerably improved compared with epirubicin.

## Patients and Methods

### Patient Population

In this retrospective study, a total of 1,471 patients with breast cancer of stages I, II, and III were enrolled at the Department of Surgical Oncology, Sir Run Run Shaw Hospital (affiliated with Zhejiang University, Hangzhou, China). The characteristics and clinical information of patients were retrospectively reviewed and stored in a regularly maintained electronic database for future reference. Among these patients, 661 diagnosed with breast cancer between 2000 and 2018 were treated with PLD as adjuvant chemotherapy; 810 diagnosed between 2001 and 2018 were treated with epirubicin as adjuvant chemotherapy. All patients had ethnicity of Han Chinese and a median follow-up time of 45.9 months. This study was approved by Ethics Committee of Sir Run Run Shaw Hospital, School of Medicine, Zhejiang University.

### Patient Treatment

All patients enrolled in this study have not received neoadjuvant chemotherapy. The radical surgery (i.e., the extensive resection of the primary tumor, together with the removal of the surrounding regional lymph nodes) to the primary breast tumor was performed before PLD and epirubicin were administered. PLD was given at 35∼40 mg/m^2^ with cyclophosphamide (CTX) every 3 weeks (4 cycles), which could be followed by docetaxel or paclitaxel every 3 weeks (4 cycles). Epirubicin was given at 90∼100 mg/m^2^ with the rest of the treatments same as PLDs. Patients were further treated with trastuzumab if the HER2 expression was positive in breast tumor. The above chemotherapy regimens were determined in terms of National Comprehensive Cancer Network^1^ guideline for breast cancer. Radiation and endocrine therapy for certain patients were also performed after chemotherapy.

### Patient Assessment

The recorded clinical information included date of diagnosis, age at diagnosis, date of recurrence and death, clinical stage, molecular subtype, tumor size, time of surgery, and menopause status. The assessment of tumor was performed with mammography, ultrasound, pulmonary CT, breast MRI, and PET-CT. The clinical stage of tumor was determined based on the American Joint Committee on Cancer (AJCC) 8th Edition.^2^ The molecular subtype included HER2-positive, luminal A/B, triple-positive, and triple-negative breast cancers (Cancer Genome Atlas Network, 2012). Toxicity was graded based on the American Society of Clinical Oncology (ASCO) standards.^3^ The patients treated with PLD or epirubicin was followed up at a 3-month interval until November 2019 and December 2019, respectively. The health status of patients was assessed at an interval of 3 months with necessary laboratory tests and imaging examinations. During the study, the left ventricular ejection fraction (LVEF) was measured with echocardiogram and electrocardiogram (ECG) was also recorded.

### Statistical Analysis

One goal of this study was to compare the efficacy between epirubicin-based and PLD-based chemotherapies, including the difference of OS and DFS. OS was defined as the time from the start of treatment (i.e., surgery) to the date of death; DFS was defined as the time from the start of treatment to the date of cancer progression. Patients still alive or without disease progressing at the end of the study were censored at the date of last follow-up. OS and DFS were analyzed using the Kaplan–Meier method, and the log-rank test was used to assess the difference between the survival curves. The Cox proportional hazards model was used to calculate the hazards ratios (HR) and corresponding 95% confidence interval (CI) for the two chemotherapies for OS and DFS. Multivariate analysis using the Cox proportional hazards model included other variables of age, menopause status, cancer stage, molecular subtype, and tumor size. Another goal was to compare the toxicity profiles between the epirubicin-based and PLD-based chemotherapies. Student’s *t*-test and chi-squared test were used to compare the side effects and the patient characteristics between the two groups. Unless otherwise specified, all statistical tests were two-sided and p-values < 0.05 were considered statistically significant. All statistical analysis was performed with the R language.^4^ All figures were prepared with the publicly available R packages (Wickham, 2009; Mailund, 2019; Wang et al., 2020).

## Results

### Patient Characteristics

Among the 1,471 patients analyzed in this study, 55.1% received epirubicin-based adjuvant chemotherapy and 44.9% received PLD-based adjuvant chemotherapy. For the two groups, the median follow-up time was 63.3 and 30.5 months, respectively. Patients in the two groups have similar menopause status (*p* > 0.05 by chi-squared test; Table 1) but slightly different age distributions (*p* < 0.05 by *t*-test, effect size Cohen’s *d* = –0.16). Patients receiving epirubicin-based chemotherapy had a higher proportion of stage II breast cancer (*p* < 0.05 by chi-squared test; Table 1) and a larger tumor size on average (*p* < 0.05 by KS test), which was adjusted in the multivariate analysis below.

**TABLE 1:** Clinical and pathological characteristics of breast cancer patients treated with PLD or epirubicin-based regimens.

Characteristic	Epirubicin (*n* = 810)	PLD (*n* = 661)	Effect size (CI)		*p*-value	Test
Age	48.9 (22.5–73.3)	50.7 (23.5–76.6)	–0.16	Cohen’s d	0.003	*t*-test
≤ 50 years	456 (56.3%)	310 (46.9%)				
> 50 years	354 (43.7%)	351 (53.1%)				
Menopause			1.1	Cliff’s Delta	0.59	Chi-square test
Yes	38.4% (311)	40.2% (266)				
No	58.0% (470)	57.2% (378)				
Missing	3.6% (29)	2.6% (17)				
Stage			0.17	Cliff’s Delta	< 0.001	Chi-square test
I-A	41.1% (333)	56.7% (375)				
II-A	41.2% (334)	30.9% (204)				
II-B	9.5% (77)	5.6% (37)				
III-A	3.3% (27)	3% (20)				
III-B	0.4% (3)	0% (0)				
III-C	0.5% (4)	0.3% (2)				
Missing	4.0% (32)	3.5% (23)				
Molecular subtype			0.1	Cliff’s Delta	< 0.001	Chi-square test
HER2-positive	12.3% (100)	7.7% (51)				
Luminal A/B	48.5% (393)	56.0% (370)				
Triple-positive	15.2% (123)	12.6% (83)				
Triple-negative	16.9% (137)	20.6% (136)				
Missing	7.0% (57)	3.2% (21)				
Tumor size	1.9 (0.09–10.7)	1.7 (0.05–11.0)	0.23	Cohen’s d	< 0.001	*t*-test

*Data are presented with median (range) for age and tumor size, and n (%) for the rest. PLD, pegylated liposomal doxorubicin.*

### Efficacy of the Two Regimens

**FIGURE 1:**
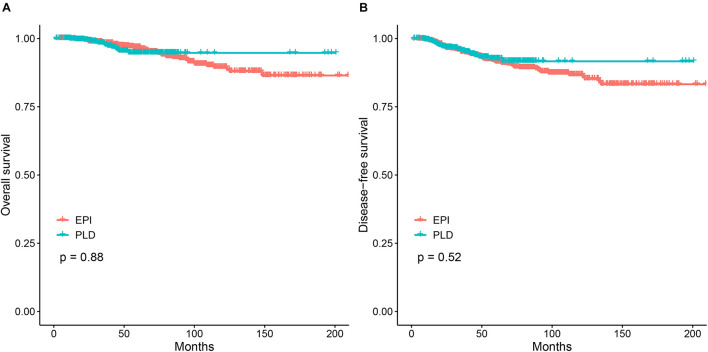
The Kaplan–Meier analysis for the PLD-based regimen vs. the epirubicin-based regimen. **(A)** Comparison of OS between the two regimens. **(B)** Comparison of DFS between the two regimens. PLD, pegylated liposomal doxorubicin; EPI, epirubicin.

The 3–, 5–, and 10-year OS were 98.0%, 94.7%, and 94.7% for PLD-based adjuvant chemotherapy and 98.2%, 96.8%, and 89.5% for epirubicin-based one. The Kaplan–Meier method showed no significant difference of OS between the two groups (*p* > 0.05; Figure 1A). Multivariate analysis using the Cox proportional hazards model showed that the HR of the PLD-based regimen was 1.02 (95% CI: 0.50–2.09) after adjusting for multiple factors (age, menopause status, stage, molecular subtype, and tumor size). Meanwhile, the 3–, 5–, and 10-year DFS were 95.5%, 92.6%, and 91.6% for PLD-based adjuvant chemotherapy and 95.0%, 91.4%, and 86.8% for the epirubicin-based one. Similar to OS, the Kaplan–Meier method did not show significant difference of DFS between the two groups (*p* > 0.05; Figure 1B) and Cox proportional hazards model yielded an unchanged HR associated with the PLD-based regimen for DFS (HR = 0.94; 95% CI: 0.57–1.56) after adjusting for the above factors.

**FIGURE 2:**
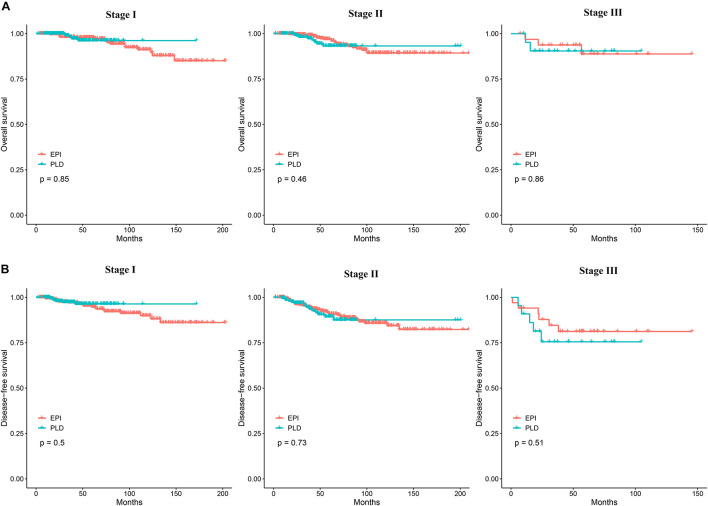
**(A)** The Kaplan–Meier curves comparing OS between the two groups for stage I, II, and III breast cancer. **(B)** The Kaplan–Meier curves comparing DFS between the two groups for stage I, II, and III breast cancer. OS, overall survival; DFS, disease-free survival.

To assess the efficacy of the two regimens for different stages of breast cancer, we performed survival analysis for patients with stage I, II, and III breast cancer. The 3–, 5–, and 10-year OS of the two regimens for different cancer stages is shown in Supplementary Table 1. The Kaplan–Meier method showed no significant difference of OS between the two regimens for each stage (Figure 2A). The Cox proportional hazards model showed that the HR of the PLD-based regimen was 0.90 (95% CI: 0.38–3.00), 1.37 (95% CI: 0.59–3.15), and 1.17 (95% CI: 0.19–7.08), respectively, for stage I, II, and III breast cancers. The 3–, 5–, and 10-year DFS of the two regimens for different stages is also shown in Supplementary Table 1. The Kaplan–Meier method showed no significant difference of DFS between the two regimens for each stage (Figure 2B). For DFS, the Cox proportional hazards model showed that the HRs of the PLD-based regimen was 0.74 (95% CI: 0.31–1.76), 1.11 (95% CI: 0.61–2.05), and 1.49 (95% CI: 0.45–4.90) respectively, for stage I, II, and III breast cancers.

**FIGURE 3:**
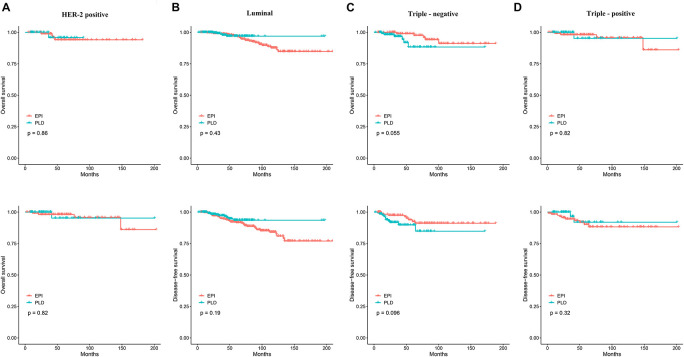
**(A)** The Kaplan–Meier curves comparing OS and DFS between the two groups in the HER2-positive patients. **(B)** The Kaplan–Meier curves comparing OS and DFS between the two groups in the luminal patients. **(C)** The Kaplan–Meier curves comparing OS and DFS between the two groups in the triple-negative patients. **(D)** The Kaplan–Meier curves comparing OS and DFS between the two groups in the triple-positive patients. OS, overall survival; DFS, disease-free survival.

We also assessed the efficacy of the two regimens for patients with molecular subtypes of HER2-positive, luminal, triple-positive, and triple-negative. For HER2-positive patients, the Kaplan–Meier method showed no significant difference in OS and DFS (Figure 3A), and the Cox proportional hazards model showed that the HR of the PLD-based regimen was 0.83 (95% CI: 0.09–7.40) and 1.25 (95% CI: 0.23–6.82) for OS and DFS, respectively. Similarly, for luminal patients, no difference in OS and DFS was observed (Figure 3B) and the HR of PLD-based regimen was 0.67 (95% CI: 0.25–1.83) and 0.63 (95% CI: 0.32–1.26) for OS and DFS, respectively; for triple-positive patients, no difference in OS and DFS was observed (Figure 3D) and the HR of the PLD-based regimen was 0.77 (95% CI: 0.08–7.38) and 0.47 (95% CI: 0.10–2.18) for OS and DFS, respectively; and for triple-negative patients, no difference in OS and DFS was observed (Figure 3C) and the HR of the PLD-based regimen was 3.30 (95% CI: 0.92–11.83) and 2.18 (95% CI: 0.85–5.57) for OS and DFS, respectively.

### Side Effects

We further compared the cardiac toxicity between the two groups. Based on available patients’ LVEF data (159 patients from the PLD-based group and 132 from the epirubicin-based group), Student’s *t*-test showed that LVEF decreased significantly less in the PLD-based group (*p* < 0.05; Supplementary Table 2). For available ECG data (626 patients from the PLD-based group and 479 from the epirubicin-based group), chi-squared test also showed that PLD-based group had more normal cases after treatment (*p* < 0.05; Supplementary Table 2). All these results suggested that the PLD-based regimen caused less cardiac toxicity.

## Discussion

Although anthracyclines have a long history of being used in adjuvant chemotherapy for breast cancer treatment, their cumulative toxicity has led to life-threatening side effects that restrict further clinical applications (Khasraw et al., 2012; Nicolazzi et al., 2018; Cai et al., 2019). For the purpose of reducing the toxicity, PLD has been developed and is now used in treating both early and advanced breast cancer (Gabizon et al., 2008; Zhao et al., 2017). Pegylated liposomal doxorubicin appeared to be an efficacious and relatively safe option as adjuvant therapy (based on 180 patients), as the toxicities associated with the treatment were mostly manageable (Lu et al., 2016). In this study, we compared survival outcomes and cardiac toxicity between PLD- and epirubicin-based adjuvant therapy for stage I–III breast cancer. We showed that PLD had similar antitumor efficacy as epirubicin while inducing lower level of cardiac toxicity, which suggests that PLD-based adjuvant chemotherapy could be a preferred treatment option for breast cancer patients especially with existing cardiac disease.

To the best of our knowledge, this study has at least the following three major strengths. First, this study has a large sample size consisting of 1,471 patients, which allows reliable statistical inference; second, this study has a relatively long follow-up time (up to 209.2 months), which is important for monitoring the cardiac side effects; third, this study has a comprehensive record of clinical information, which makes it possible to compare OS and DFS in patient subgroups (i.e., different stages and molecular subtypes).

One obvious limitation is the retrospective design of this study. Although over 1,400 patients with breast cancer were analyzed in this study, lack of prospective randomization could make our results less convincing. Another limitation is that all patients enrolled in this study (at Sir Run Run Shaw Hospital) were Han Chinese, which makes the conclusions potentially inapplicable to other ethnicity groups even in China. In addition, PLD-based regimen needs to be compared with well-performing non-anthracycline regimens for breast cancer treatment in China (Jones et al., 2006). Nevertheless, this study warrants large-scale prospective studies with more ethnicity groups enrolled in the near future.

## Conclusion

By comparing the efficacy and toxicity profiles between PLD- and epirubicin-based adjuvant therapy for stage I–III breast cancer, we conclude that, in Han Chinese, PLD has a similar anti-breast cancer efficacy as epirubicin while it induces a lower level of cardiac toxicity. This study also suggests that, for breast cancer patients (especially those with existing cardiac disease), PLD-based adjuvant chemotherapy should be a better option than the epirubicin-based one.

## Data Availability Statement

The data analyzed in this study is subject to the following licenses/restrictions: The patient data analyzed in this study are not publicly available due to the confidentiality of the data but are available from the corresponding author on reasonable request. Requests to access these datasets should be directed to WH, wenxianhu@zju.edu.cn.

## Ethics Statement

The studies involving human participants were reviewed and approved by Ethics Committee of Sir Run Run Shaw Hospital, School of Medicine, Zhejiang University. Written informed consent for participation was not required for this study in accordance with the national legislation and the institutional requirements.

## Author Contributions

WH designed the overall project, analyzed the data, and wrote the manuscript. KL and RT analyzed the data. JC and CX collected clinical information of patients. LJ and YC assisted in data analysis. WZ assisted in manuscript preparation. All authors read and approved the final manuscript.

## Conflict of Interest

The authors declare that the research was conducted in the absence of any commercial or financial relationships that could be construed as a potential conflict of interest.

## Publisher’s Note

All claims expressed in this article are solely those of the authors and do not necessarily represent those of their affiliated organizations, or those of the publisher, the editors and the reviewers. Any product that may be evaluated in this article, or claim that may be made by its manufacturer, is not guaranteed or endorsed by the publisher.

## Footnotes

1
www.nccn.org/patients

2
https://cancerstaging.org/

3
https://www.asco.org/

4
http://www.r-project.org/

## Abbreviations

PLD: pegylated liposomal doxorubicin
OS: overall survival
DFS: disease-free survival
LVEF: left ventricular ejection fraction
ECG: electrocardiogram
EPI: epirubicin
CI: confidence interval
HR: hazard ratio
CTX: cyclophosphamide
AJCC: American Joint Committee on Cancer.
